# Dynamic Interaction of Spindles and Gamma Activity during Cortical Slow Oscillations and Its Modulation by Subcortical Afferents

**DOI:** 10.1371/journal.pone.0067540

**Published:** 2013-07-02

**Authors:** Miguel Valencia, Julio Artieda, J. Paul Bolam, Juan Mena-Segovia

**Affiliations:** 1 Neurophysiology Laboratory, Neuroscience Area, Centro de Investigacion Medica Aplicada, Universidad de Navarra, Pamplona, Spain; 2 Medical Research Council Anatomical Neuropharmacology Unit, Department of Pharmacology, University of Oxford, Oxford, United Kingdom; University of Alberta, Canada

## Abstract

Slow oscillations are a hallmark of slow wave sleep. They provide a temporal framework for a variety of phasic events to occur and interact during sleep, including the expression of high-frequency oscillations and the discharge of neurons across the entire brain. Evidence shows that the emergence of distinct high-frequency oscillations during slow oscillations facilitates the communication among brain regions whose activity was correlated during the preceding waking period. While the frequencies of oscillations involved in such interactions have been identified, their dynamics and the correlations between them require further investigation. Here we analyzed the structure and dynamics of these signals in anesthetized rats. We show that spindles and gamma oscillations coexist but have distinct temporal dynamics across the slow oscillation cycle. Furthermore, we observed that spindles and gamma are functionally coupled to the slow oscillations and between each other. Following the activation of ascending pathways from the brainstem by means of a carbachol injection in the pedunculopontine nucleus, we were able to modify the gain in the gamma oscillations that are independent of the spindles while the spindle amplitude was reduced. Furthermore, carbachol produced a decoupling of the gamma oscillations that are dependent on the spindles but with no effect on their amplitude. None of the changes in the high-frequency oscillations affected the onset or shape of the slow oscillations, suggesting that slow oscillations occur independently of the phasic events that coexist with them. Our results provide novel insights into the regulation, dynamics and homeostasis of cortical slow oscillations.

## Introduction

Slow oscillations (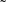
0.8 Hz) are a widespread phenomenon among sleeping mammals. They emerge in the deeper phases of sleep, extending over large cortical areas [1], [2], and conserve their fundamental properties during anesthesia [3] and in isolated cortical preparations [4]. At the cellular level, slow oscillations are generated by fluctuations in the membrane potential of pyramidal neurons in cortical layers 2/3 and 5. Such fluctuations produce two distinctive states: a depolarized state, where neuronal activity tends to occur (UP state), and a hyperpolarized state, where neuronal activity tends to be absent (DOWN state) [5].

One of the distinctive features of the slow oscillations is the presence of spindles and high frequency oscillatory activity (gamma, 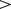 20 Hz) during cortical UP states [6]. Spindles are produced as a consequence of low-threshold Ca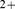 spikes from hyperpolarized neurons in the reticular thalamic nucleus at the onset of the UP state [7], presumably triggered by the input from pyramidal neurons [8]. In turn, reticular thalamic neurons recruit and synchronize thalamocortical neurons that will produce a feed-forward loop onto pyramidal neurons [9]. After a few cycles, spindles wane as a consequence of an increased desynchronization in cortical discharge [10]. On the other hand, gamma activity emerges in neocortical networks as a consequence of either local connectivity or interactions with thalamocortical neurons [11].

Spindles and gamma activity are critical for the homeostatic regulation of sleep and have been associated with cognitive processes during sleep [12]–[14]. For example, spindle density during sleep is increased in rats and humans following reward learning [15], declarative tasks, and procedural motor skills [14], particularly in areas activated during the encoding of the task, suggesting a long-term potentiation of synapses involved in the previous behavior. In contrast, the association of gamma with cognitive functions during sleep is less clear. It has been reported that coherence in different bands, including gamma, increases following learning performance [16].

Here we analyzed the electrocorticogram of anesthetized rats during slow oscillations to understand how low- and high-frequency oscillations interact and give rise to the characteristic patterns of slow wave activity. We observed that neither spindles nor gamma activity have any effect on the duration of the UP states, nor do they trigger the onset of the slow oscillation cycle. In addition, we also tested the effects of brainstem activation on the dynamics of oscillations. We detected a high level of interdependency between the different frequency oscillations that is susceptible to disruption by ascending activating mechanisms in a frequency-specific manner.

## Materials and Methods

### Ethics Statement

All experiments were performed in accordance with the Animals (Scientific Procedures) Act, 1986 (UK) and the Society for Neurosciences policy on the use of animals in neuroscience research, under the Project License 30-2639 approved by the Home Office and the local ethical committee of the University of Oxford.

### Electrophysiological Recordings and Local Drug Application

Experiments were carried out on 7 adult male Sprague-Dawley rats (250–310 g; Charles River, Margate, UK). Anesthesia was induced with 4% v/v isoflurane in 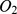, and maintained with urethane (1.3 g/kg, i.p.), and supplemental doses of ketamine (30 mg/kg, i.p.) and xylazine (3 mg/kg, i.p.), as previously described [17]. Electrocorticographic activity and reflexes were monitored to ensure the animals' well-being and depth of anesthesia.

The electrocorticogram (ECoG) was recorded via a 1 mm diameter steel screw juxtaposed to the dura mater above the frontal cortex (3.0 mm anterior and 2.5 mm lateral of bregma [18]), which corresponds to the somatic sensorimotor cortex [19], and referenced to a steel screw located above the cerebellum. Signals were filtered at 0.3–1000 Hz, amplified 2000-fold and digitized on-line at 2.5 kHz. Activity was recorded during slow-wave activity (SWA; defined broadly in the delta range: 0.5–4 Hz) which accompanies deep anesthesia and is similar to activity observed during natural sleep. All biopotentials were digitized on-line using a Power1401 analog-to-digital converter (Cambridge Electronic Design) and stored in a personal computer running Spike2 acquisition and analysis software (Cambridge Electronic Design).

For local drug application, glass pipettes (tip diameter 20–40 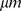) were filled with saline solution (0.9% w/v NaCl) containing an acetylcholine receptor agonist, carbachol (20 mM), and inserted into the brain under stereotaxic guidance. Small volumes were slowly infused (100 nl over 30 s, total dose: 2 nmol) into the right pedunculopontine nucleus (PPN) and their effects on the ipsilateral ECoG activity were quantified (see below).

Recordings were acquired during epochs of robust and stable cortical SWA (pre-carbachol) and immediately after the infusion of carbachol (post-carbachol). After reviewing the recordings, for every animal and condition (pre- and post-carbachol) at least 200 s segments (mean: 301.64 s, SD: 34.79) of continuous recordings were selected for further analysis.

### Spectral Analysis

Welch periodograms (window size: 4 seconds, overlap: 80%, Hanning window, resolution of 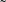
0.1 Hz per bin) were used to compute the power spectral density of the ECoG. These power spectral density estimations were then used to calculate band power (i.e. mean power of all frequencies within the band) for three frequency bands: slow (0.3–3 Hz, SO), alpha (6–15 Hz; referred to as ‘spindles’) and gamma (30–70 Hz) oscillations.

### SWA Characterization

**Figure 1:**
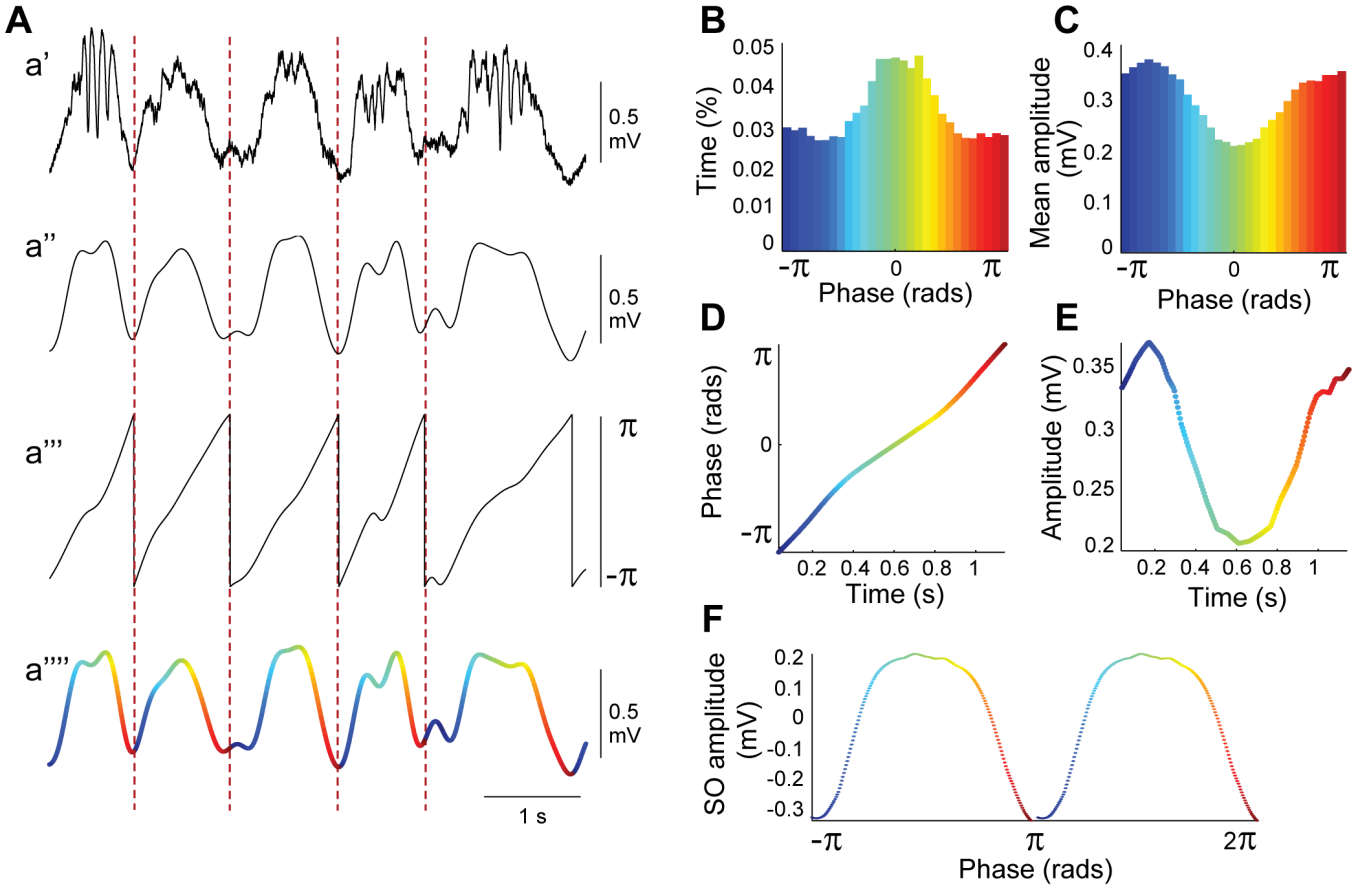
Slow oscillation phase analysis. A, Extraction of the slow oscillation phase from a raw ECoG trace during SWA: a’, Raw ECoG trace during SWA; a”, the same signal is filtered in the slow oscillations range (0.3–3 Hz); a”’, the Hilbert transform is used to obtain the phase of the slow oscillation; a””, the phase of the slow oscillation is then color-coded from 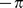 (dark blue) to 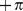 (red) passing by 0 radians (green) over the temporal evolution of the slow oscillation. B, C, Percentage of time spent and mean amplitude of each of the 30 phases in which the slow oscillation (SO) was divided. D, E, Time course of the phase and amplitude dynamics across one slow oscillation cycle. F, Slow oscillation mean waveform across a five-minute recording was obtained in each animal to identify the mean amplitude, mean duration and the phase distribution (two cycles are displayed). The same color code as in a”” was used for panels B–F.

Stages in the SWA were detected automatically using custom MATLAB routines based on the instantaneous phase of the slow oscillations, 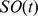 (Fig. 1). Raw signals were bandpass filtered between 0.3–3 Hz (finite impulse response filter with no phase delay, filter 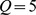) and the instantaneous phase and amplitude of the resultant slow oscillations were determined from its analytic representation 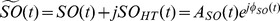, where 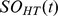 is the Hilbert transform, 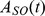 is defined as the instantaneous amplitude or envelope of the oscillation and 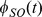 is called the instantaneous wrapped phase as it is constrained to an interval 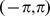 and represents the fraction of one complete wave cycle.

For a perfect sinusoidal signal with central frequency 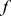, phase evolves linearly from 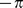 to 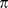 with a period of 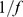. Typically for slow oscillations, the phase evolves more slowly around UP states than in the vicinity of DOWN states (the UP and DOWN state nomenclature is only used descriptively in this manuscript as quantification is not possible). Dividing the 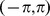 phase interval into 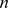 phase-slots, allows the determination of how long the wave remains in each of these specific phase-slots and thus allows determination of a continuum of phase-states that characterizes the oscillation. Again, for the case of the perfect sinusoidal signal, the time spent in each phase portion equals 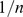 thus giving a uniform distribution. In contrast, in the case of slow oscillations where UP states are longer than DOWN states, the times spent on phase slots around 0 radians are longer than those close to 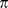. Taking into account the whole recording and averaging the time lengths corresponding to each phase-slot gives an estimation of the percentage of time spent by the waves in each of the phase intervals (here we used 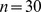 phase intervals). Figure 1B shows the average time distribution for the slow activity of a representative animal showing a non-uniform distribution with longer times located around 0 radians (peak of the slow activity, i.e. UP state). Similarly, the distribution of the mean amplitudes (envelopes) for each phase-slot can be obtained (Fig. 1C). By linear interpolation it is possible to change from the phase to the time domain and to recover patterns for the average phase 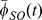 and amplitude 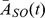 of one typical cycle of slow oscillation that accounts for the whole recording (Figs. 1D and E, respectively). Then, the shape of this typical cycle can be computed by taking the real part of the resultant pattern: 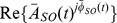.

Oscillations in the spindle and gamma-frequency bands were extracted using bandpass filters between 6–15 and 30–70 Hz respectively (finite impulse response filter with no phase delay, filter 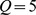) and amplitude envelopes were obtained by means of the Hilbert transform as described above. Then, the distribution of the amplitude and energies (i.e., the square of the amplitude values) for each phase slot across the slow oscillation cycle was obtained.

To analyze the probability of occurrence of fast frequency oscillations, we computed the median amplitude of oscillations as the median amplitude envelope after bandpass filtering (same parameters as those described above) and Hilbert transform, and used this as the threshold for defining the existence of an active oscillation (an oscillation above the noise level) [20]. For each of the 30 phase intervals across the slow oscillation cycle, four categories were considered: 1) intervals with spindles only, 2) intervals with gamma oscillations only, 3) intervals with both spindles and gamma, and 4) intervals with neither spindles nor gamma. Then the probability for each of these categories was computed. For the recordings after carbachol administration, the threshold was defined from the basal condition (as described above).

### Cross-frequency Coupling (CFC)

Cross-frequency interactions between different frequency bands were assessed by means of the modulation index (MI), as outlined by Tort [21]. First, raw data is filtered in two frequency ranges of interest. The instantaneous phase of the slow wave (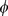) and the amplitude (envelope) of the fast oscillation (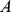) are then computed by means of the Hilbert transform as explained above. Finally, the coupling between the phase of the slow wave and the amplitude of the fast activity is determined by the entropy of the distribution obtained by computing the mean amplitude of the instantaneous envelope for 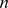 intervals of the instantaneous phases. A normalized entropy index is computed 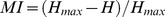 where 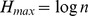 is the maximum entropy 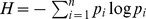 and 
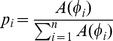
 is the normalized amplitude of the fast oscillation at each of the 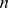 phase intervals considered (here we set 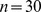). When the MI is 0, there is no phase-to-amplitude modulation and the phase-amplitude distribution is uniform, whereas larger MI values arise from stronger modulation. Assessment of the statistical significance of the MI value is obtained by computing 200 surrogates by adding a temporal random offset to the amplitude signal. Then a 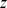-score of the strength of phase to amplitude coupling 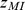, can be computed.

Here we analyzed the cross-frequency coupling between slow oscillations and fast frequencies (spindles and gamma) and between spindles and gamma oscillations. For the coupling between the slow oscillations and fast frequencies, we first calculated the phase signal by filtering raw data from 0.2 to 3.5 Hz (defining 50 finite impulse response filters with no phase delay, central frequencies linearly distributed and 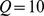) and the amplitude signal filtering from 5 to 100 Hz (50 finite impulse response filters with no phase delay, central frequencies linearly distributed and 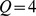).

Once we observed that the phase of the slow oscillations determined the amplitude of both, spindles and gamma activities, we examined which was the preferred SO-phase for these increases in amplitude of the fast activities. Thus, we used 30 phase bins for the SO phase (extracted after bandpass filtering between 0.3–3 Hz, 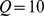 and Hilbert transform) and studied the distribution of the mean amplitude of the instantaneous envelope for each of them (50 finite impulse response filters with no phase delay, central frequencies linearly distributed and 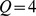). The coupling between spindles and gamma oscillations was analyzed by computing the phase signal between 6 and 16 Hz (50 finite impulse response filters with no phase delay, central frequencies linearly distributed and 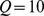) and the amplitude signal from 25 to 100 Hz (50 finite impulse response filters with no phase delay, central frequencies linearly distributed and 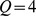). The “referred spindle-phase” for the gamma oscillations was determined by filtering the phase signal in the 6–15 Hz interval (finite impulse response filter with no phase delay, 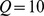). The analysis of the coupling between spindles and gamma oscillations was constrained only to those periods where active spindle oscillations were detected.

To analyze the dynamics of the gamma oscillations in relation to the evolution of the spindles, we evaluated the flow of the gamma energy from the onset of the spindles at the beginning of the UP state through 8 consecutive cycles. To achieve this, we first defined the onset of the spindle as the first cycle whose amplitude exceeded the threshold (determined by the median of the spindle energy) in the vicinity of the transition from the DOWN to the UP state. Then, we computed the unwrapped phase for each detected spindle from its onset 8 cycles forward and 4 backward. As a result, the phase of each spindle is obtained in the interval 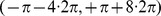, with phase value equal to 0 corresponding to its onset. By dividing each 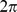 radians segment (i.e., one complete cycle) in 30 equal intervals, it is possible to obtain the amplitude of the spindles and energy of the gamma oscillations evolving across 13 consecutive cycles. Averaging these values for the whole set of detected spindles allows the recovery of the shape of a “mean spindle” and a “mean gamma time course” associated with the evolution of the spindle.

### Constrained Cross-frequency Coupling cCFC

To evaluate whether the spindle-gamma coupling is different as a function of the phase of the slow oscillation, we extended the MI concept from two to three signals and defined a constrained cross-frequency coupling index, 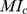. The phase of the slow oscillations 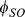 and spindles 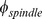 were extracted and divided into 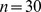 and 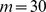 intervals respectively. Then, we evaluated the mean instantaneous amplitude of the gamma oscillations 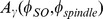, for each 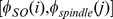 interval with 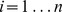 and 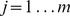. Finally, the constrained modulation index 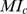 can be estimated by means of 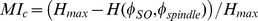 where 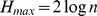 and 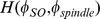 represents the entropy of a bidimensional variable: 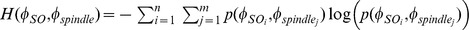 where again, 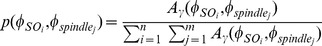 is the normalized amplitude of the gamma oscillation in each of the 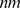 phase bidimensional intervals considered.

### Statistics

Data are expressed as means and standard error of the means (SEM) or standard deviation (SD). The effects of carbachol on the spectral content of the SWA were assessed by means of a cluster-based, non-parametric permutation paired test. Additionally, differences in the pre-post carbachol energy values for each of the three selected frequency bands (slow oscillations, spindles and gamma oscillations; mean power of all frequencies within the band) were assessed by means of paired t-test. Paired t-tests were also used to assess differences in the average duration and amplitude of the slow oscillation before and after carbachol administration.

Prior to any statistical analysis, values were normalized using the transformation described by van Albada and Robinson [22]. This transformation is based in the fact that a uniform distribution can be obtained from any continuous variable by computing its cumulative distribution function (CDF). So, the normalization of non-gaussian variables can be reached by the application of a function (inverse error function) that transforms a uniform distribution to a normal distribution.

Cluster-based nonparametric permutation paired-tests [23] were used to assess the effects of carbachol administration on the shape of the slow oscillation amplitude and phase patterns, on the power changes in the spindles and gamma oscillations across the slow oscillation cycle and also on the probability of occurrence for these fast activities.

Carbachol effects on the interaction between frequencies were studied in different ways. First, we used cluster-based nonparametric permutation paired tests again to compare the distributions of the mean amplitude of the instantaneous envelope across the slow oscillation cycle. Then, we used paired t-tests to compare the pre/post MI values (previously normalized) for the slow oscillation/spindles, slow oscillation/gamma and spindles/gamma oscillations pairs. Finally, effects of carbachol on the preferred phase and degree of modulation were assessed by means of bivariate-paired Hotelling's T-square tests. Differences in the pre/post carbachol constrained cross-frequency coupling MI values were assessed by means of paired t-tests, while differences in the cross-frequency coupling pattern 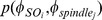, were studied with the use of non-parametric permutation test over the 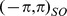, 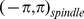 plane.

## Results

### Slow Wave Activity Configuration

**Figure 2:**
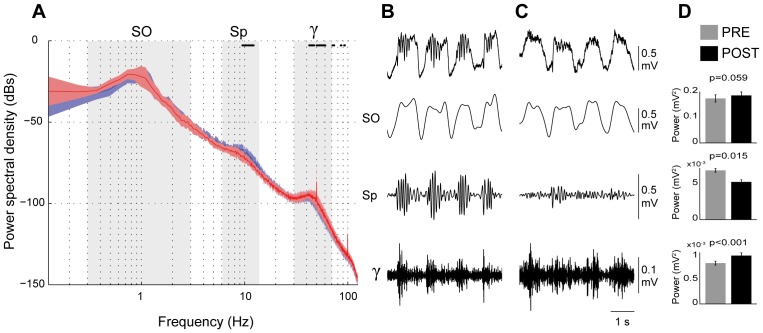
Cortical slow wave activity decomposition. A, Power spectral density (PSD) analysis identifies three frequency bands during slow wave activity before (blue trace) and after carbachol (red trace) injection: slow (SO; 0.3–3 Hz), spindles (sp; 6–15 Hz) and gamma (30–70 Hz) oscillations (black dashes denote frequency bins where significant differences between conditions were found, 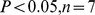). B, Raw ECoG traces and their corresponding decomposition for the predominant three bands mentioned above. Cortical UP states are defined by the largest energy in the slow oscillation band that correlate with increases in the amplitude of spindles and gamma activity. The episodic increases in the amplitude in both frequency bands are coupled to the slow wave activity. C, D, Following a carbachol injection into the PPN, there is a significant decrease in the spindle amplitude (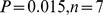) and an increase in the gamma band energy (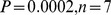), while the slow oscillations maintain their temporal structure and have a tendency to increase their energy (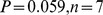).

We recorded the ECoG activity from the frontal cortex of urethane- and ketamine/xylazine-anesthetized rats during stable cortical SWA. SWA was characterized by a prevalent slow rhythm in the 0.3 to 3 Hz frequency range. Periods of light cortical activation (lower amplitude and higher frequency oscillations) spontaneously alternated with SWA, as described previously for urethane recordings [24], [25]. Similar to sleep recordings and recordings from anesthetized animals [6], three predominant frequency oscillations were observed following power spectrum decomposition (Fig. 2A): slow oscillations (0.3 to 3 Hz, with a peak at around 1 Hz), spindles (6 to 15 Hz, with a peak at around 9 Hz) and gamma oscillations (30 to 70 Hz, with a peak at around 40 Hz; Fig. 2B). As described previously [6], [25], the phasic increases in the amplitude of faster oscillations (spindles and gamma) coincided with the peak of amplitude in the slow-oscillation cycle (equivalent to the UP state). Conversely, the lowest amplitude in spindle and gamma activity was correlated with the troughs of the slow oscillations (equivalent to the DOWN state). Across individual recordings, the associated higher-frequency components of the slow oscillations varied from cycle to cycle, indicating unique oscillatory signatures for each slow oscillation cycle, similar to the organization and oscillatory patterns observed during sleep in humans [26].

### Carbachol Effects on the Energy Balance of Cortical Oscillations

Previously we reported that the injection of a low carbachol dose (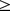 2 nmol) into the pedunculopontine nucleus (PPN) during cortical slow oscillations increased the power of gamma oscillations that was confined to the duration of cortical UP states [25]. Here we detected that carbachol produced significant differences in the spectral content of the SWA in specific frequency points within the spindles and gamma ranges (Fig. 2A, cluster-based, non-parametric paired permutation test, 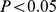). Additionally, we evaluated the effects of the carbachol in the PPN on all three predominant frequencies during cortical SWA (i.e., slow oscillations, spindles and gamma oscillations; Fig. 2C, D). We observed a robust increase in the power of the gamma oscillations (16.91%; post/pre ratio: 1.1691 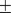 0.0324 SEM; paired t-test after normalization 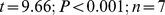) with a significant decrease in the power of the spindles (22.58%; post/pre ratio: 0.774 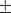 0.0789 SEM; paired t-test after normalization 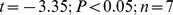) when compared to the recordings immediately before the injection. The power of the slow oscillations showed an increase that did not reach significance levels (7.5%; post/pre: 1.0749 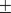 0.0395 SEM; paired t-test after normalization 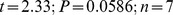).

### Temporal Coincidence of Spindles and Gamma Activity during Slow Oscillations

**Figure 3:**
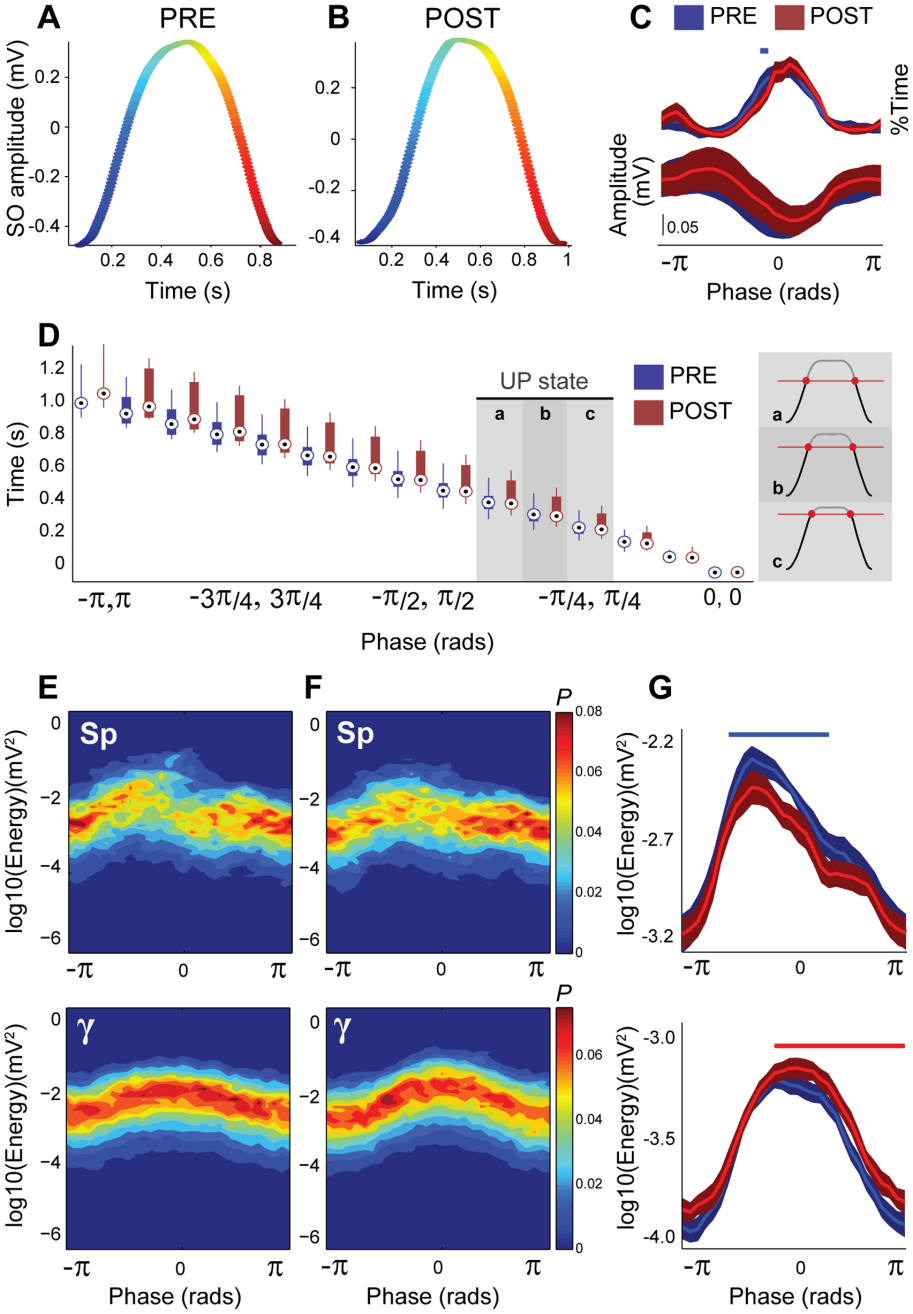
Carbachol affects the energy balance of the slow wave activity. A, B, Slow oscillation mean waveform before and after a carbachol injection into the PPN (slow oscillation phases are color-coded as in Fig. 2) for a representative animal. C, No major structural changes were observed as a result of the drug, as measured by the amplitude of the slow oscillation and the time spent in each phase, and only two phase points (out of 30 evaluated) were significantly different after carbachol (horizontal line), revealing a decrease in the time of the transition from DOWN state to UP state of the slow oscillation (n = 7). D, Boxplot showing the duration of the transition times taken by the slow oscillation to go from 15 different negative phase points (ranging from -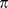 to 0) to their corresponding (symmetric) positive values (from 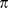 to 0). As a reference, the line on top of three pair of values shows a series of putative UP states detected according to the values −1.2566 (a), −1.0472 (b) and −0.8377 (c) rads, and as depicted in the schematic oscillations in the inset (red lines represent the three phase values in a, b and c). No effects of carbachol on the duration of putative UP states nor in any of the other durations were detected. E–F, Probability distribution of the spindle and gamma amplitudes (log-transformed) for each of the 30 phases in which the slow oscillation cycle was divided, before and after the carbachol injection. E, The spindle amplitude was largest in the transition from the DOWN state to the UP state, and then decreases towards the center of the UP state (upper panel). In contrast, the highest gamma amplitude coincides with the center of the UP state (lower panel; warm colors represent higher energy). F, G, As a result of the carbachol injection, the spindle energy during the DOWN-to-UP transition decreases, but towards the second half of the UP state the differences are no longer significant (upper panels). Carbachol increases the gamma energy at the point of its highest amplitude and during the up-to-down transition (lower panels). Horizontal lines in G indicate significant differences.

In order to characterize the organization of the different frequencies that compose SWA, we first obtained information on the mean duration and amplitude of slow oscillations across animals (n = 7), and observed that the slow oscillation cycle typically has an average amplitude of approximately 0.8 mV (mean: 0.767, SD: 0.196 mV) and average duration of approximately 1s (mean: 0.975, SD: 0.098 s) (Fig. 3A). Following the carbachol injection, the overall structure remained very similar (Fig. 3B, mean duration of the SO cycle: 1.0466, SD: 0.144 s; amplitude: 0.791, SD: 0.190 mV) and no significant differences in the duration (paired t-test after normalization 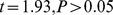) and amplitude (paired t-test after normalization 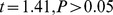) of the slow oscillation cycles between both conditions were observed (Fig. 3C). However, when we tested differences across the 30 phases into which the slow oscillation cycle was divided, we observed a significant decrease in the percentage of time spent in the transition from DOWN state to UP state after carbachol injection (cluster-based nonparametric permutation paired test, 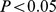; Fig. 3C). To further test the potential effect of carbachol on the duration of the putative UP states, we compared the average time spent across the 30 phase points described above using symmetrical ranges (i.e., from 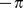 to 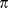, then from 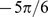 to 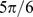, and so on until 0) (Fig. 3D; only 15 phase points are depicted). We chose three putative markers of the UP state based on the waveform of the slow oscillation and found no significant differences in the time spent between these ranges (note that no differences were observed between any ranges of the slow oscillation cycle, including DOWN states; paired t-test after normalization, 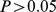). These data suggest that carbachol infused into the PPN does not have any effect on the duration of the UP states.

We then evaluated the dynamics of the faster frequency oscillations (i.e., spindles and gamma) across the slow oscillation cycles to detect possible differences in their organization or distribution. Spindles showed their highest energy at the beginning of the UP state and then tended to decrease progressively. In contrast, gamma oscillations were higher at the peak of the slow oscillation (zero phase), coinciding with the UP state (Fig. 3E, G). After the carbachol injection, the spindles energy decreased significantly (cluster-based nonparametric paired permutation test, 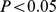) during the first half of the UP state, whereas the gamma energy increased in the second half of the UP state (Fig. 3F, G). These data suggest that spindles and gamma activity have a distinct temporal organization within the slow oscillation cycle.

**Figure 4:**
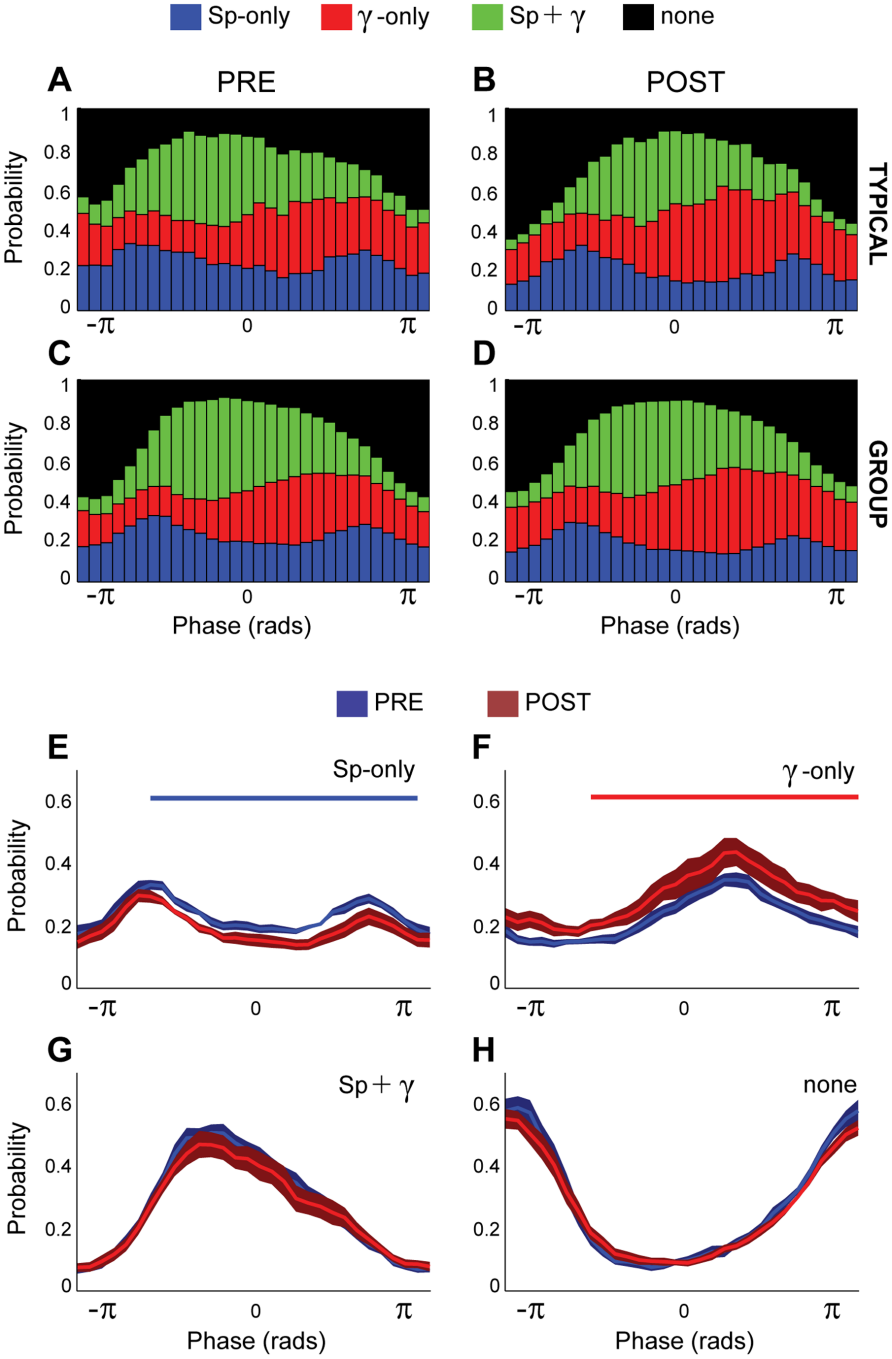
Co-occurrence of spindles and gamma activity. A–D, Probability of emergence of spindles and gamma activity for each of the 30-point phases that compose the slow oscillation cycle, before (A, C) and after (B, D) carbachol injection, for a single case (A, B) or the group total (C, D) (n = 7). Four possible scenarios were considered: the presence of spindles-only (blue), the presence of gamma-only activity (red), the co-occurrence of spindles and gamma activity (green), or the absence of any of the above (black). E–H, The differences in the emergence probabilities for each of these scenarios were compared before and after the carbachol injection. Thus, the probability of the emergence of gamma-only activity increases significantly following the carbachol injection, whereas the probability of spindles-only diminishes. Horizontal lines in E and F indicate significant differences. No differences in the probability of spindles-gamma co-occurrence or absence of these frequencies were observed. Note that the bimodal distribution of the spindle-only episodes can be explained by the increase in the probability of emergence of the co-existing spindle-gamma episodes at the middle of the UP state. Data are expressed as mean and standard error of the mean (SEM).

We next investigated whether spindles and gamma activity coexist during slow oscillation cycles. We evaluated the probability of occurrence of each of the following combinations and analyzed in four categories: 1) slow oscillation intervals (i.e., each of the 30 phase fragments in which the slow oscillation cycle has been divided) with spindles alone (‘spindles-only’), 2) slow oscillation intervals with gamma alone (‘gamma-only’), 3) slow oscillation intervals with both spindles and gamma (‘spindle-associated gamma’), and 4) slow oscillation intervals with neither spindles nor gamma (Fig. 4). ‘Spindle-only’ activity shows a bimodal distribution in the slow oscillation cycle, with a higher probability of emergence at the beginning and at the end of the UP state (Fig. 4A, C). In contrast, ‘gamma-only’ activity shows a higher probability to be present at the end of the UP state. During the first half of the UP state, the probability of spindles and gamma activities occurring together is higher than the probabilities of each of them alone. During the DOWN state, the probability of spindles and gamma is much lower than the probabilities of ‘spindles-only’ or ‘gamma-only’, suggesting that the coexistence of spindles and gamma is more representative of the UP state. Finally, the probability of the absence of spindles and gamma or their combination, is progressively lower towards the center of the UP state and higher during the DOWN state.

Following the carbachol injection, no qualitative changes in the pattern of distribution of spindles and gamma within the slow oscillation cycle were observed (Fig. 4B, D): spindles showed the same bimodal distribution, gamma showed more probability of occurrence at the end of the UP state, and the probability of co-occurrence of spindles and gamma was higher during the UP state and lower during the DOWN state. Nevertheless, statistical analysis detected that the probability of ‘spindles-only’ intervals decreased significantly (cluster-based nonparametric paired permutation test, 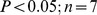) following the carbachol injection affecting the majority of the slow oscillation cycle, although the onset of the first peak of emergence of spindles, coincident with the beginning of the UP state, was not modified (Fig. 4E). In contrast, the probability of ‘gamma-only’ intervals significantly increased (cluster-based nonparametric paired permutation test, 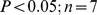) and this effect was most prominent after the onset of the UP state and at a time point after the drop of spindles probability (Fig. 4F). The probability of ‘spindles-gamma’ intervals was not modified between conditions (Fig. 4G), nor was the probability of either of the above combinations (Fig. 4H). Thus, these results show that spindles and gamma activities on their own have a distinct distribution within the slow oscillation cycle, and their co-existence occurs at different phases of the cycle and is not affected by an increased subcortical activation produced by the carbachol injection.

### Spontaneous and Carbachol-driven Interactions between Frequencies during Cortical SWA

**Figure 5:**
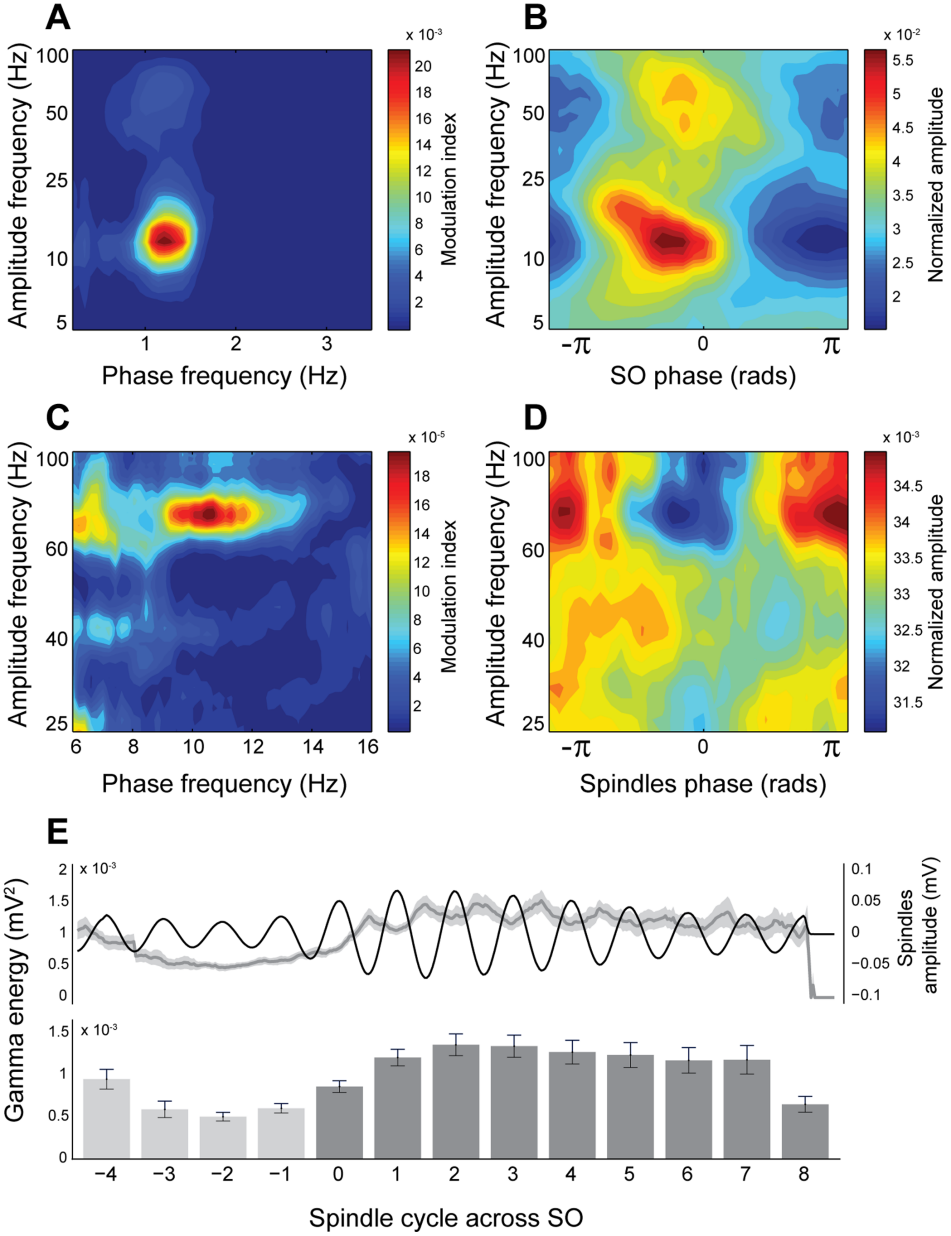
Cross-frequency coupling during SWA. A, Comodulogram in the ranges 0.2–3.5 and 5–100 Hz for one representative animal, revealing that the phase of the slow oscillations modulates the amplitude of spindles and gamma oscillations; no other frequencies are modulated during SWA. B, Distribution of the normalized mean amplitude (between 5 and 100 Hz) across the phases of one slow oscillation cycle for the same animal. Spindles and gamma activity show a distinct temporal distribution in their coupling to the slow oscillation cycle. C, Same representation as in A but for the spindle phase (6–15 Hz interval), revealing that the amplitude of gamma is also coupled to the spindles. D, Distribution of the normalized mean amplitude (25–100 Hz) for each phase of the spindles, showing that the highest gamma amplitude is coupled to the trough of the spindle cycle. E, Evolution of the gamma energy in relation to the temporal evolution of the spindles. Time courses have been computed by averaging spindle amplitude and gamma energy within 8 consecutive spindle cycles (see methods). The upper traces show the time course of the mean spindle and the mean gamma energy computed for each cycle. The bar plot shows the mean energy within each spindle cycle and shows an increase in the gamma energy with the appearance of the first spindle cycle (cycle 0; dark gray bars) and a slight decrease onwards from the second/third cycles. Over this slow dynamic, a faster modulation of the gamma energy is also observed. Note that transient increments of the gamma energy are coincident with spindle troughs while transient decrements are detected in the vicinity of the spindle peaks (n = 7).

Once we determined that spindles and gamma activity co-exist within slow oscillations, we tested whether there is any interaction between these oscillations. We analyzed the cross-frequency coupling between the phase of the slow oscillations and the amplitudes of the spindle and gamma activities to determine whether there is any dependency of fast frequencies on the slow frequencies. We observed that the phase of the slow oscillation significantly determined the amplitude of both spindles (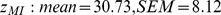) and gamma activities (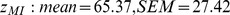) and that no other frequencies were coupled to the slow oscillation (see Fig. 5A for a representative case). The normalized amplitude of spindles and gamma oscillations across the slow oscillation cycle revealed that the amplitudes of the spindles and gamma were maximal at different phases of the slow oscillations, with spindles coupled mainly to the first half of the UP state and gamma aligned to the center of the UP state (Fig. 5B). Notably, both frequencies of oscillation showed coincident coupling to the slow oscillation just before the center of the UP state. This distribution coincides in time with the maximal probability of emergence of slow oscillation cycles with both spindles and gamma, as previously shown (spindle-associated gamma, Fig. 4).

Because spindles and gamma oscillations co-exist and their occurrence overlaps during part of the slow oscillation cycle, we tested whether there is an interaction between these two frequencies of oscillation. Using the spindle phase frequency in the range of 6–15 Hz, the cross frequency coupling analysis revealed that the amplitude of gamma depends on the phase of the spindles (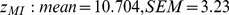) predominantly in the 9–12 Hz range (see Fig. 5C for a representative case). In this case, the distribution of the normalized amplitude of the gamma oscillation shows that the highest amplitude in gamma is localized in the troughs of the spindle cycle (−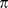, +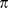), and conversely, the lowest amplitude in gamma coincides with the spindles' peak (zero phase; Fig. 5D). Even though the strongest amplitude of the gamma oscillations was localized in the low gamma range (30–60 Hz), in some cases the coupling also spread to the high gamma range (60–100 Hz; note that the coupling value of frequencies around 50 Hz is underestimated due to interference with the AC power supply artifact). Thus, these data show that when spindles and gamma co-exist, the amplitude of gamma depends on the phases of both activities, slow oscillations (coupled to the peak) and spindles (coupled to the trough).

Once we determined that spindles and gamma are functionally coupled, we then evaluated the dynamics of gamma over the evolution of the spindles from their onset at the beginning of the UP state (Fig. 5E). As described above, on average spindles showed their largest amplitude at the onset of the UP state, followed by a gradual reduction across approximately 8 cycles. In contrast, the energy of spindle-associated gamma showed its maximal values towards the third spindle cycle, suggesting that the peak amplitude of gamma is shifted from the peak amplitude of the spindles. These results suggest two distinct dynamics in spindle-associated gamma: fast dynamics associated with the spindle waveform where increases in gamma energy are linked to the trough of the spindles, and slow dynamics over the course of several spindle cycles.

**Figure 6:**
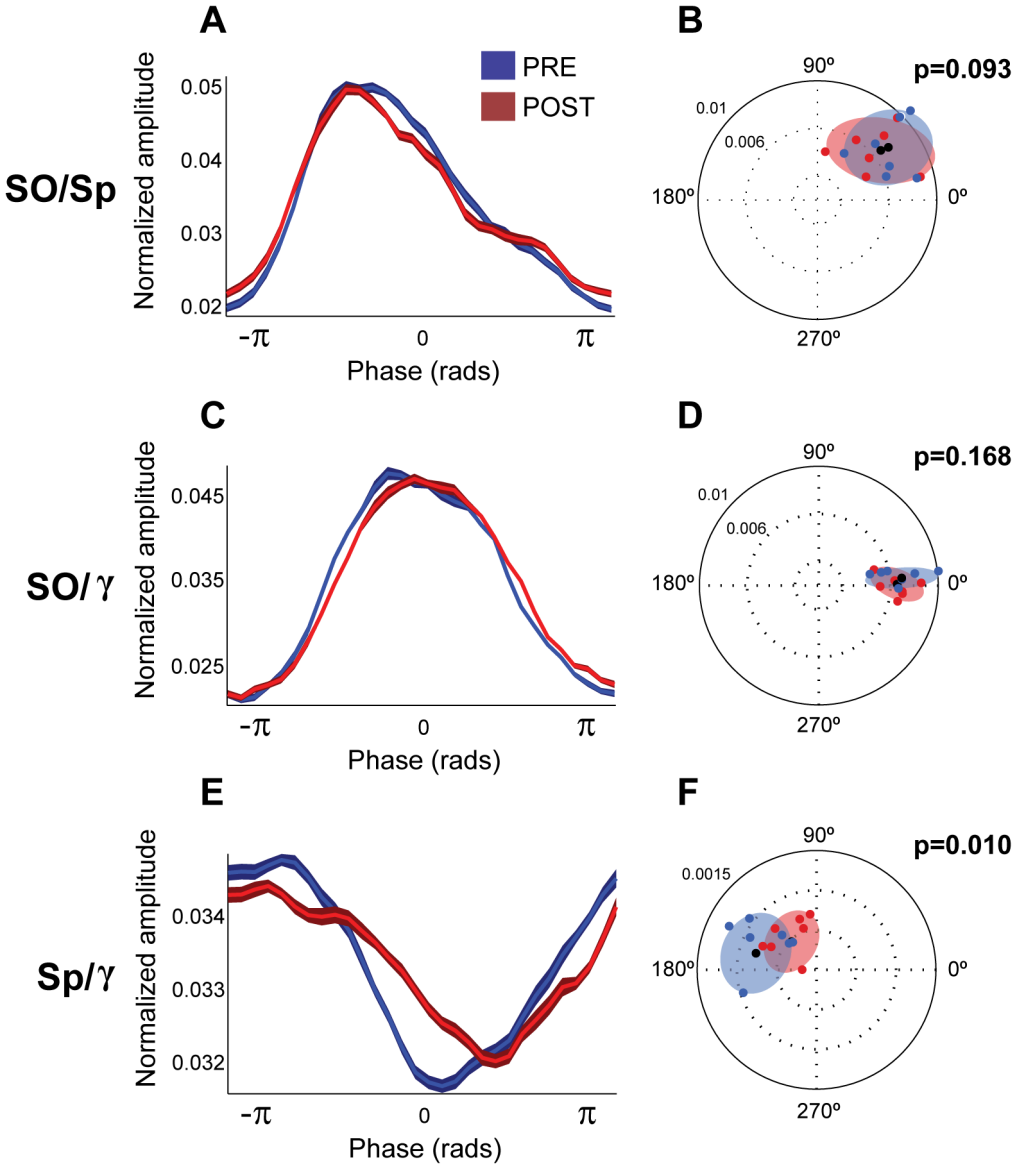
Carbachol effects upon frequency interactions. Phase-to-amplitude modulation between slow oscillations and spindles (A,B), slow oscillations and gamma (C,D), and spindles and gamma (E,F), before (blue) and after (red) a carbachol injection into the PPN (n = 7). The distribution of the normalized mean amplitude of spindles (A) and gamma (C) in relation to the slow oscillation phase, and gamma in relation to the spindle phase (E), show that, while the coupling of spindles or gamma to the slow oscillations does not vary across the cycle, the coupling of gamma to the spindle cycle is shifted in time. While carbachol preserves the modulation of the slow oscillation over the spindles and gamma amplitude, it has an effect in the strength of modulation for the spindles-gamma pair. This effect becomes significant when the phase shift is taken into account only for the spindles-gamma modulation (F), which is not observed on the spindles (B) and gamma (D) modulation. Blue/red dots in panels B, D and F represent the resultant of the normalized amplitude (taking into account phase and amplitude values) for each animal and condition, shaded areas represent the minimum ellipsoid enclosing the data points (n = 7) for each condition and black dots are the centroids of each ellipsoid, thus representing the point of strength and phase of modulation for each population. P-values correspond to the significance of the bivariate-paired Hotelling's T-square tests.

Once we characterized the interaction between the distinct frequencies that compose the SWA, we tested the effects of the increased subcortical tone on this interaction by carbachol stimulation of PPN output. We calculated the distributions of the normalized amplitudes for each of the three pairs of interactions described above: slow oscillations and spindles (Fig. 6A, B), slow oscillations and gamma (Fig. 6C, D), and spindles and gamma (Fig. 6E, F). The amplitude of the spindles that are dependent on the phase of the slow oscillations maintained the same shape between conditions (i.e., amplitude of spindles remains at maximum at the onset of the UP state; Fig. 6A) and no differences in the modulation index (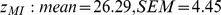) nor in the preferred-phase/modulation depth index were detected (paired t-test after normalization 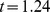, P = 0.263 and bivariate-paired Hotelling's T-square tests 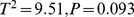 respectively, see Fig. 6B). Similarly, the amplitude of gamma that is dependent on the slow oscillations remained coupled to the peak of the UP state after the carbachol injection (Fig. 6C), and the modulation index (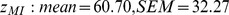) and preferred-phase/modulation depth index (Fig. 6D) were not different between pre and post carbachol (paired t-test after normalization 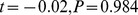 and bivariate-paired Hotelling's T-square tests 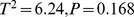, respectively). In contrast, the amplitude of gamma that depends on the spindles phase was shifted and the lowest gamma amplitude was no longer situated at the trough of the spindle cycle (Fig. 6E). The modulation index tended to decrease following the carbachol injection (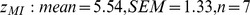) but this effect did not reach significance (paired t-test after normalization 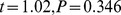). However, when both preferred-phase and modulation depth were considered, statistical tests (bivariate-paired Hotelling's T-square tests 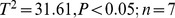; Fig. 6F) detected that the coupling pattern was significantly different as a result of the drug injection, suggesting that carbachol selectively disrupts the coupling between spindles and gamma.

**Figure 7:**
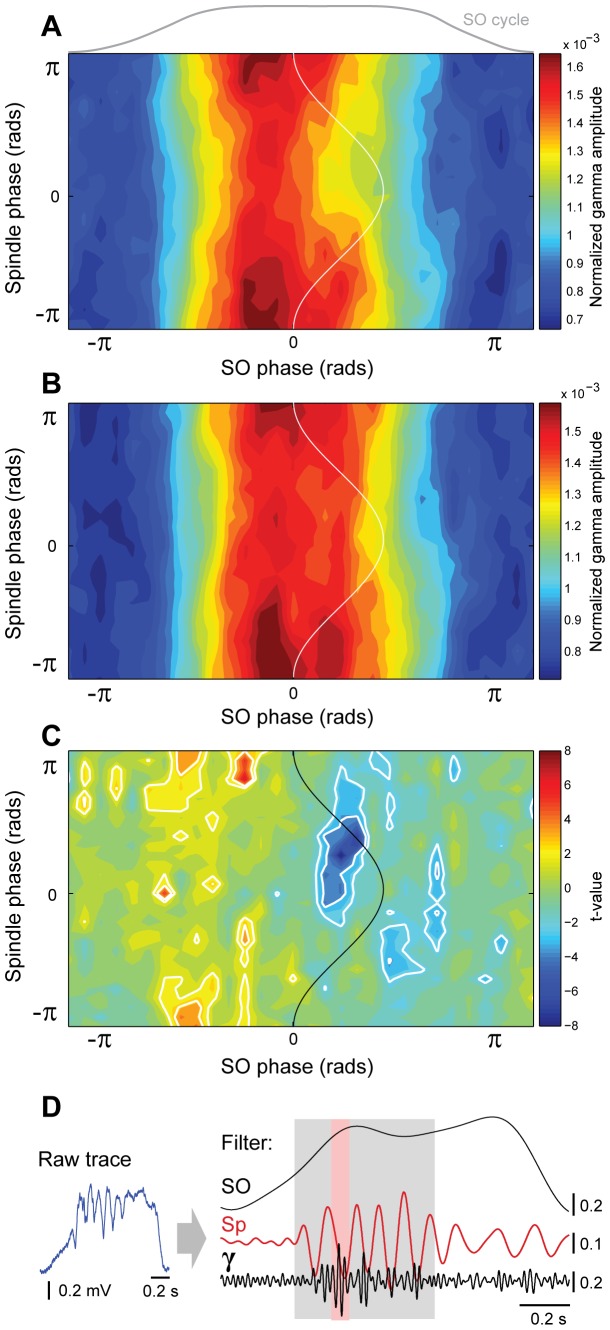
Interaction between spindles and slow oscillations determines the gamma energy. Constrained cross-frequency coupling (cCFC) during SWA before (A) and after (B) the carbachol injection, and the statistical differences between conditions (C). Normalized gamma amplitude (represented as warm colors in A and B) has a strong dependency on the slow oscillation phase, and a weaker dependency on the spindle phase. Statistical analysis revealed discrete differences in these patterns as a result of the experimental manipulation. White lines define areas showing significant increases (red, 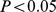 and 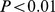) and decreases (blue) in the distribution of the gamma amplitudes. Differences are mainly found at the trough of the spindle cycle at the beginning and end of the cortical UP state. D, Raw data illustrating the temporal organization of the three oscillation frequencies within one representative slow oscillation cycle. Spindles and gamma occur during the gray-shaded area of the slow oscillation cycle, whereas gamma in turn occurs during the red-shaded area of the spindle cycle. Vertical scale bars represent mV.

Finally, because the gamma amplitude depends on the phase of both slow oscillations and spindles, we evaluated whether the spindle-gamma coupling is different as a function of the phase of the slow oscillation. A constrained cross-frequency coupling analysis that considered the phase of spindles and the phase of the slow oscillations as determinants of the gamma amplitude, reveals that the deepest spindle-gamma modulation is located immediately before the peak of the slow oscillation, and shows that in the second half of the UP state such modulation depends on the spindle phase (Fig. 7A, D), reflecting the low gamma coupling to the peak of the spindles shown in Fig. 5D. The same constrained cross-frequency coupling analysis after the carbachol injection (Fig. 7B) did not show any effects on this phenomenon and no significant differences in the constrained cross-frequency coupling index 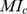, were detected (paired t-test after normalization 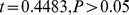). Nevertheless, point-to-point analysis (paired t-test after normalization, 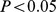) of the normalized amplitude of the gamma oscillation pattern 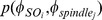, shows that the spindles-gamma modulation shifts in relation to the slow oscillation phase, decreasing during the onset of the UP state and extending towards the end of the UP state, homogenizing the gamma amplitude across the spindles cycle and therefore decreasing the modulatory drive of the spindle phase (Fig. 7C). These data show that carbachol produces a decoupling of gamma from the spindles while still preserving its modulation by the slow oscillations.

## Discussion

We analyzed the slow wave activity in the frontal cortex of rats under anesthesia and observed the same frequency components previously reported in rodents and other species, including humans, during natural sleep: slow oscillations, spindles and gamma. We observed that UP states determine the appearance of the spindles and gamma activity, but in turn the phasic increases of these faster frequencies do not have an impact on the duration of the slow oscillations or their onset. In addition, we found that spindles and gamma activity coexist during the UP state of cortical slow oscillations, that they have distinct dynamics across the slow oscillation cycle, and that they are functionally coupled at precise time points of the UP state. We then examined the effects of an increased subcortical drive by the activation of an arousal-related structure, the pedunculopontine nucleus (PPN), and observed that the amplitude and emergence probability of gamma oscillations was increased, whereas the same measures were reduced for spindles. In addition, we detected significant changes in the functional coupling between spindles and gamma. Our results provide fundamental insights into the composition and dynamics of the slow oscillations, and provide a detailed framework to understand the contribution of subcortical structures to the regulation of cortical oscillations during slow wave activity.

### Slow Oscillation Architecture

The origin of the cortical slow oscillations is determined by the synchronization of multiple cortical neurons whose membrane potentials fluctuate and alternate between hyperpolarized (DOWN states) and depolarized (UP states) configurations [27]. While cortical interneurons and pyramidal neurons are sufficient to provide the onset of the slow oscillations, the duration of the cycle seems to be dependent on both intrinsic mechanisms and thalamocortical inputs that shape the slow oscillation [4], [28]. The synchronized depolarization and neuronal activity during UP states is accompanied by the presence of high frequency oscillations (spindles and gamma) nested within the 1 Hz rhythm, but it remains unclear whether their presence has any effect on the architecture of the slow oscillation. Here we show that gamma activity has no effect on the duration of the slow oscillations, or specifically the UP states, but tends to increase their amplitude, although the effect was not significant. On the other hand, the decrease in the amplitude of the spindles produced a change in the shape of the slow oscillation at the point where the highest amplitude of the spindles tends to occur (the onset of the slow oscillations), rather than a direct effect on their duration. Thus, such reduction in the amplitude of the spindles did not significantly affect the overall duration of the slow oscillations, suggesting that the phasic increases in the spindles at the onset of the slow oscillations do not trigger the slow oscillation cycle. Altogether, our results suggest that the high frequency oscillations that emerge during the cortical UP states do not determine the appearance, amplitude or duration of the slow oscillations. Our data then supports the evidence that cortical slow oscillations are a cortically-sustained mechanism [4], [27].

The analysis of the structure, organization and distribution of high frequency oscillations reveal that spindles and gamma coexist and interact during cortical UP states, supporting findings from sleeping humans [29], [30]. While the origin of spindles has been consistently associated with the activity of the reticular thalamic nucleus, gamma oscillations appear to have at least two distinct origins. Thus, cortical circuits intrinsically generate gamma oscillations during cortical UP states when the cortex is isolated in brain slice preparations [31]. On the other hand, subcortical structures are also considered to contribute to this activity through thalamic structures of diffuse cortical projection (e.g., intralaminar thalamic nuclei) [11]. Here we demonstrate that by modifying the output of the PPN, an ascending modulatory structure that innervates several thalamic nuclei, we are able to increase the energy in the gamma frequency range and increase the probability of emergence of gamma oscillations in the slow oscillation cycle. Our results are thus in line with the notion of a subcortical source of gamma activity and suggest an interplay between the locally-generated and the subcortically-driven gamma activity.

### Subcortical Modulation of Cortical Oscillations during SWA

Increasing evidence supports the notion of organized subcortical activity during SWA. Distinct structures in the midbrain and brainstem with upstream projections to thalamic and cortical targets remain active during cortical slow oscillations and their activity is highly structured. Such is the case of the pedunculopontine nucleus [25], [32], the locus coeruleus [33] and the raphe dorsalis [34], among others (for a review see [35]). Furthermore, a recent fMRI human study in naturally sleeping subjects was able to confirm that regions in the brainstem were active during cortical slow oscillations [36]. We have reported that identified cholinergic neurons in the PPN fire preferentially during the UP state of cortical slow oscillations and are timed with phasic increases in gamma activity [25]. Because their local axon collaterals make synaptic contacts in the PPN, we hypothesized that the local release of acetylcholine by PPN neurons, timed with the changes in the amplitude of cortical gamma, would be functionally correlated. We then showed that carbachol injections into the PPN increased gamma oscillations during the UP state of cortical slow oscillations, demonstrating that ascending pathways arising in the PPN are able to affect gamma oscillations during SWA. In the present study we proposed to understand how the slow oscillations are composed and the role of the subcortical structures in shaping cortical activity during SWA. Using a similar experimental approach we analyzed the whole frequency spectrum to understand the consequences of such activation. Our data revealed that carbachol produces a specific increase in ‘gamma-only’ activity with a consequent decrease in ‘spindle-only’ activity. Occurrence of slow oscillations together with ‘gamma-only’ activity increased significantly their probability of emergence, whereas ‘spindle-only’ activity decreased their probability of appearance. These results suggest that ‘gamma-only’ activity has a subcortical origin and is modulated by structures like the PPN that are able to have an impact on its gain.

In contrast to the above, the co-occurrence of slow oscillations and ‘spindle-associated gamma’ activity was unchanged. This suggests that the activation of subcortical structures, driven by the carbachol injection, has an impact on ‘gamma-only’ activity, but not on gamma activity that is associated with the spindles. We also revealed that ‘spindle-associated gamma’ depends on the phase of the spindles, indicating a functional dependence. We did not observe any effect of carbachol on the probability of emergence of ‘spindle-associated gamma’ or its energy, as observed in the difference between pre- and post-carbachol for the gamma energy (see Fig. 7C). The lack of effect on ‘spindle-associated gamma’ energy contrasts with the robust increase in energy of the ‘gamma-only’ episodes. However, we detected a decrease in the modulation index associated with a shift in the phase coupling to the spindles. This means that ‘spindle-associated gamma’ becomes more homogeneous across the spindle cycle after receiving a carbachol injection, or in other words, gamma decouples from the spindles. We interpret this finding as a consequence of the negative modulation of the spindles rather than an effect on the spindle-associated gamma. Together, these data suggest that the ‘spindle-associated gamma’ has a different origin to the ‘gamma-only’ activity induced by carbachol, supporting the idea that ‘spindle-associated gamma’ has a local (cortical) origin and it is likely to be structured by reticular thalamic inputs through the modulation of spindles.

### Functional Significance

Spindles and gamma activities are hallmarks of the cortical slow oscillations, and together are hallmarks of slow wave sleep. Recent evidence shows that both spindles and gamma play an important part in the functional role of the slow oscillations and have been associated with plasticity and learning processes [12], [15]. Nevertheless, the mechanisms for the regulation of the phasic components of the slow oscillations have not been completely elucidated. Our data show that subcortical structures play a part in the regulation of SWA by modulating gamma oscillations during cortical UP states. It is not clear whether intrinsic cortical circuits are able to increase the gain following the demands brought by plastic changes, but here we provide evidence that such gain could be provided by subcortical circuits of ascending control. This supports the notion of a gating mechanism at subcortical levels that could be modulated depending on the cognitive demand. Recent evidence shows that this mechanism is present in locus coeruleus neurons [33]. Thus, new experiences or new learning could potentially increase the activity of ascending neuromodulatory neurons and have an impact on the dynamics and functional interactions of fast frequencies during SWA. Our data demonstrate that these phenomena of oscillatory interactions occur in a model of sleep (anesthesia) and provide insight into the mechanisms of regulation and functional significance of cortical slow oscillations that also occur during natural sleep.
